# Concept of an Intervention for Sustainable Weight Loss in Postmenopausal Women with Overweight—Secondary Analysis of a Randomized Dietary Intervention Study

**DOI:** 10.3390/nu15143250

**Published:** 2023-07-22

**Authors:** Isabell Englert, Sarah Egert, Laura Hoffmann, Kathrin Kohlenberg-Müller

**Affiliations:** 1 Department of Nutritional, Food and Consumer Sciences, University of Applied Sciences, 36037 Fulda, Germany; laura.hoffmann@oe.hs-fulda.de (L.H.); kathrin.kohlenberg-mueller@oe.hs-fulda.de (K.K.-M.); 2 Department of Nutrition and Food Science, Nutritional Physiology, University of Bonn, 53113 Bonn, Germany; s.egert@uni-bonn.de

**Keywords:** overweight, weight loss, individual nutrition, group sessions, postmenopausal women

## Abstract

Introduction: The approach of an individual diet has great potential for sustainable weight reduction. Social support, participation and empowerment are also key factors for high motivation and compliance. So, the impact of an individual diet in combination with group sessions on weight loss in postmenopausal women with overweight was investigated. Methods: In this reanalysis of a controlled intervention study, postmenopausal women (*n* = 54; BMI 30.9 ± 3.4 kg/m^2^; 59 ± 7 years) were recruited receiving an energy restricted diet for 12 weeks, followed by a six-month follow-up phase. The women received 51 individual meal plans based on their habits and were trained in four group sessions. Results: Forty-six women completed the intervention phase, and 29 completed the follow-up. Average weight loss was −5.8 ± 3.0 kg (*p* < 0.001) after 12 weeks and was still significant at follow-up (−4.9 ± 5.4 kg, *p* < 0.001). Also, decreases in fat-free mass (−1.1 ± 1.2 kg, *p* < 0.001) and resting energy expenditure (−1096 ± 439 kJ/24 h, *p* < 0.001) were observed. Conclusions: The individual nutrition approach with a focus on nutritype in combination with group sessions was effective for long-lasting weight loss in postmenopausal women. An important factor is close individual and group support.

## 1. Introduction

In Germany, 61% of middle-aged women (50–59 years) are overweight, and 27% are obese; the prevalence of overweight and obesity rises with age [1]. Although excess body weight is associated with an increased risk of various noncommunicable diseases, such as diabetes mellitus type 2, coronary heart disease, stroke and cancer [2,3], weight loss in older adults risks the loss of lean mass [4] and the emergence of sarcopenia [5]. This risk is increased in women since they experience a higher prevalence of both obesity, due to hormonal changes, for example [6], and sarcopenia [7]. Nonetheless, intentional weight loss can improve physical function [8,9] and metabolic health [8,10,11].

Weight loss interventions show very large interindividual variation in their efficacy [12]. To achieve successful, sustainable weight reduction, the implementation method used in the intervention is crucial. In the context of public health nutrition, dietary recommendations ensure the needs of the entire population are met. However, the “one-size-fits-all” approach does not always show satisfactory results in terms of behavior change and weight loss. As a result, it is becoming increasingly important to tailor recommendations and strategies to the individual’s needs [12,13,14,15], an approach termed personalized nutrition (PN). So far, there is no uniform definition of PN; however, genotype, enterotype, metabotype and nutritype characteristics are all considered in PN recommendations [16]. These considerations encompass two factors: the biological characteristics (genotype, enterotype and metabotype) and individual nutritional preferences (nutritype) [17]. The latter also includes the social environment, cooking competence and preferences for and aversions to certain foods. Despite increasing interest in the interactions between genotype, enterotype, metabotype and nutrition, there is currently no evidence that shows that the consideration of biological factors improves the effectiveness of PN with respect to genotype [16]. The largest European study on PN so far, the 6-month, randomized controlled Food4Me trial with 1269 participants, showed that, compared to not receiving PN recommendations, PN resulted in an improved diet (as measured by a healthy eating index). However, targeting PN to the current diet was only equally as effective as targeting PN to the current diet plus phenotype or current diet plus phenotype and genotype [18]. These results need to be verified.

In parallel, process-driven actions are currently being implemented in nutrition counselling to emphasize individual needs and preferences [19,20]. According to process models, an individual dietetic assessment and continuous monitoring, both of which include diet-related data, are essential [21]. Health promotion focuses on the individual’s empowerment, with interventionists aiming to “help people gain control over their own lives” [22] and help them to make healthy decisions. Simultaneously, participatory approaches are needed to maintain motivation and ensure exchanges between peers, which can contribute to higher motivation and compliance. Street at al. [23] emphasize in their recent review the need for further studies on this topic.

Therefore, the aim of this secondary analysis of a randomized dietary intervention study was to determine whether an individual approach emphasizing the nutritype, in combination with group sessions, resulted in successful weight loss in postmenopausal women with overweight. We have previously described the effects of protein intake on the preservation of fat-free mass (FFM) [24]. Our major findings were that an energy-restricted high protein diet does not preserve FFM and REE in postmenopausal overweight women. A range of subjects’ baseline parameters are represented here to better relate them with the newly presented data.

## 2. Materials and Methods

### 2.1. Trial Design and Participants

The ProSeni (Protein Intake for Seniors) study is a two-arm, randomized, double-blind controlled study for weight reduction in postmenopausal women (absence of menstruation for at least 12 months) with overweight (Supplementary Material, Figure S1). An individual approach was used, with primary and secondary outcomes assessed before (t_0_) and after the intervention period of 12 weeks (t_1_). This included four nutrition training sessions and telephone interviews after training to promote compliance and motivation, which was followed by 6-months of ad libitum food intake without intervention (follow-up, t_2_). The design and detailed methods have been previously published [24]. Briefly, fifty-four women were recruited and randomized in a 1:1 ratio to a normal protein or a protein-enhanced weight loss group. The intervention comprised individual meal plans based on a healthy western diet, with two out of at least three daily meals replaced by formula diets. The primary outcome was FFM; secondary outcomes were resting energy expenditure (REE), physical function and weight loss. The ethics committee of the Fulda University of Applied Sciences approved the study protocol, and the study is registered in the German Clinical Trial Register (DRKS00011238).

### 2.2. Intervention

The study protocol was based on the recommendations of Bellg et al. [25] for enhancement of treatment fidelity. The strategies for high study fidelity were divided into five categories: study design, staff training, delivery of treatment, receipt of treatment and enactment of treatment.

An individual’s goal was to lose, on average, 500 g of body weight per week over a period of 12 weeks, as planned by the body weight planner [26]. Therefore, the diet was energy-restricted by 3139 kJ (750 kcal) per day compared with the individual’s total energy expenditure, calculated as the measured REE (by indirect calorimetry) multiplied by the estimated physical activity level (PAL). The dietetic assessment included a detailed diet history at baseline, which was structured to ask about the meal and snack patterns in the daily routine, and preferences for and aversions to different food groups. The individually prescribed energy intake was used to create a meal that ensured weight reduction including consumption of a shake (Precon shake; Darmstadt, Germany; 1565 kJ (374 kcal), 12.2 g fat, 8.7 g carbohydrates and 46.6 g protein per 100 g) prepared with 300 mL of milk (1.5% fat) twice a day. In addition, a meal (usually warm) could be chosen from the individual diet plan. For snacking, only raw fruits and vegetables were allowed. To achieve the calculated daily energy intake and increase motivation, small desserts were included when necessary or desired. The dishes were prepared at home by the women using the recipes. Any changes to the recipes by the women were noted in the protocols. There were no guidelines regarding the timing of meal intake. Based on diet history, participants were given the first 21 individual meal plans, after which, based on the monitoring of changing preferences and aversions, participants received 10 new plans at the following training sessions. This allowed changes to be made based on individual preferences during the intervention phase, e.g., flavor of shakes, variety of food and cold or hot dishes. Monitoring of the process-guided approach occurred in the form of a food diary for 7 consecutive days after the first and third training sessions and food checklists on the remaining days; in addition, documentation lists were checked, and follow-up phone calls were made. An example diet plan is given in Table 1.

The intention of the intervention period was to empower the women to establish healthy eating habits, and thus, the learning goals “understand” and “apply” were strictly followed in all training sessions performed by nutritionists. The learning goals of the first training session were to understand the study design and to transfer the study information into practice; the individual diet plans were explained, and questions were answered. The women were empowered to apply the advice they received in their daily life via simple implementation tips and open discussion. Under active participation, women were trained in how to keep a food diary and food checklists by using a flipchart. The learning goal of the second training day focused on understanding a generally healthy diet; details have been described previously [24]. Afterwards, participants were able to identify the healthiest products within the separate food groups. Sugar and fat were the main topics in the third session: women were taught how to understand the ingredients shown on food packaging and were given a handout with a food exchange table for energy reduction to practice the learned lessons. Understanding the basics about food and emotions was the focus in the last training session. In addition, the staff used “favorite recipes” such as Spaghetti Bolognese and showed how to prepare them in a healthier way, and popular nutrition myths were discussed. Each day of training was designed to give participants time and space to share their experiences with each other (according to the “analyze” learning goal). The women were able to ask questions and share their concerns, wishes and suggestions throughout the training session. Additionally, they received their new meal plans and shakes, which also served to ensure their presence at the sessions; where this was not possible, individual appointments were arranged.

### 2.3. Follow-Up

After the intervention, the learned behaviors were to be consolidated in a 3-month follow-up period. For this, they practiced the procedure of a healthy diet based on German nutritional recommendations [27] in an individual face-to-face session. The pyramid was available as a large poster with small food cards, with which the women were asked to practice the correct “ticking off” of consumed meals. In this way, they were able to easily check what had already been eaten and in which areas foods were still needed. Furthermore, they received a protocol for beverages, a handout containing the learned information, and instructions on how to replace the shakes with suitable meals, as well as tips on how to integrate exercise into everyday life. The diet history taken at the beginning of the intervention was also discussed and beneficial pre-intervention behaviors were reinforced.

Three months later, a follow-up telephone interview was carried out concerning the following topics: current weight, past and current goals and dietary patterns (i.e., if shakes were still consumed).

Women had the opportunity to contact study staff by phone or in person with questions or problems throughout the intervention and follow-up periods; this was explicitly communicated. Motivational interviewing was practiced at all times to deal with perceived drawbacks and find individual solutions together.

### 2.4. Anthropometry and REE

Anthropometry and REE measurements were conducted by a trained nutritionist at baseline (t_0_), after the intervention time (t_1_), and after the follow-up (t_2_). REE was measured under standardized conditions [28] after an overnight fast in the morning using indirect calorimetry (Quark RMR; Cosmed, Germany). Our own a priori tests of the Quark RMR showed a reliability of r = 0.86 (*p* = 0.005). Body height was determined barefoot in a standing position using a stadiometer (seca 274; seca GmbH, Hamburg, Germany). Body weight, FFM and fat mass (FM) were recorded using bioelectrical impedance analysis (seca medical body composition analyzer; seca mBCA 515/514). Waist circumference and blood pressure (Boso medicus family, Vienna, Austria) in accordance with international guidelines and recommendations [29,30] were also measured.

Target weight loss was calculated using the body weight planner [26] based on weight, sex, age, height, PAL, fat mass (%), energy from carbohydrates (in energy%) and REE and compared with the actual weight loss for evaluation of compliance.

### 2.5. Statistics

Statistical analyses were performed using the IBM SPSS statistical software package (version 23, IBM, Armonk, NY, USA). Sample size estimation was based on an effect size of 0.69 and an SD of 1.5 as described previously [24]. Data are presented as means ± SD. Per-protocol analyses for the participants who completed the study were conducted. Within-subject analyses were used to measure effects over time (dependent *t* test, t_0_–t_1_, t_1_–t_2_). A stepwise multiple regression analysis was performed to test which variables have an influence on the loss of the FFM. Correlations were used to analyze any interrelationships. Statistical significance was defined as *p* ≤ 0.05, and all *p*-values listed are two-tailed.

For the following analysis, the data of the whole cohort were analyzed. This was feasible because in the original study there was no influence of protein intake on the study groups. Thus, the relevant outcome variables were not different.

## 3. Results

In total, 54 women participated in the study. Eight women dropped out during the intervention phase because of adverse events not related to this study (*n* = 5) and a lack of acceptance of the shakes (*n* = 3). Twenty-nine women completed the study-inclusive follow-up. Participant characteristics are shown in Table 2.

**Figure 1:**
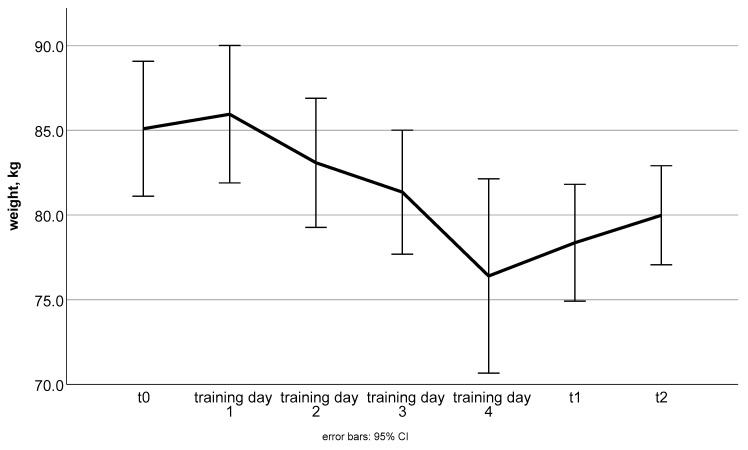
Mean weight changes with 95% confidence interval in kg over the intervention and follow-up phase.

Participants achieved significant weight loss after 12 weeks of intervention (Table 2, comparison t_0_–t_1_ and Figure 1), which was still significant after the follow-up period (t_0_–t_2_: −4.9 ± 5.4 kg; *p* < 0.001; data are not presented in Table 2). The range of weight loss during the intervention period was −14 kg to 0.3 kg; however, when outliers (values further than ±2 SD from the mean) were excluded (*n* = 2), this was reduced to 11.2 kg.

FFM, FM, and skeletal muscle mass were significantly lower after the weight loss intervention (t_1_) than at baseline (all *p* < 0.001; Table 2). At follow-up, FM showed a small but significant increase from baseline (*p* = 0.041; Table 2). The waist circumference of participants was significantly lower after the intervention (t_1_, *p* < 0.001) and was still significantly lower after the follow-up period. Overall, 37% achieved a waist circumference <88 cm at t_2_ compared to 10.9% at t_0_. The significant reduction observed in systolic and diastolic blood pressure at t_1_ (both *p* < 0.001) was reversed at t_2_, resulting in a significant increase (both *p* < 0.05; Table 2).

**Figure 2:**
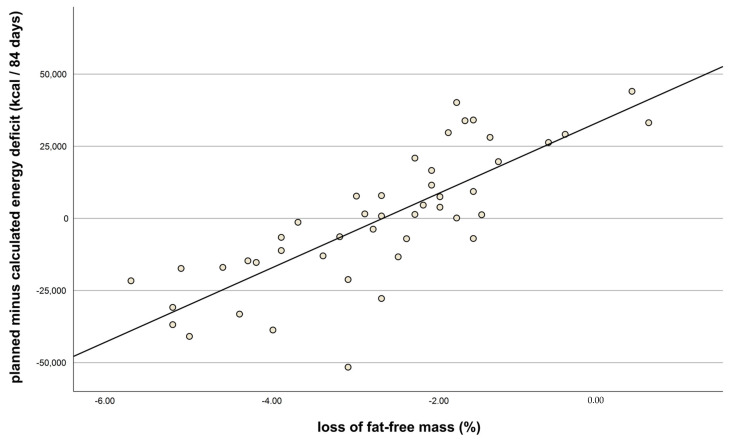
Loss of fat-free mass after intervention (%, t_1_) vs. planned minus calculated energy deficit (kcal) after 84 days (kcal), as indicated by food diaries. Correlation coefficient: r = −0.8; *n* = 46; *p* < 0.001.

A higher deficit in energy intake was associated with a greater loss of FFM (r = −0.8; *p* < 0.001, *n* = 46; Figure 2). In addition, a calculated energy deficit was significantly correlated with a loss of muscle mass (r = 0.378, *p* = 0.014, *n* = 42). Regression analysis indicated that 39% of the variance in FFM loss was explained by the amount of weight lost during the first three weeks and the energy deficit. REE was significantly lower after the weight loss intervention but significantly higher at follow-up (both *p* < 0.05; Table 2). Planned (by body weight planner) minus calculated (by food diaries) energy deficit and weight loss were both significantly positively correlated with REE loss (r = 0.39, *p* = 0.007 and r = 0.34, *p* = 0.011, respectively).

**Figure 3:**
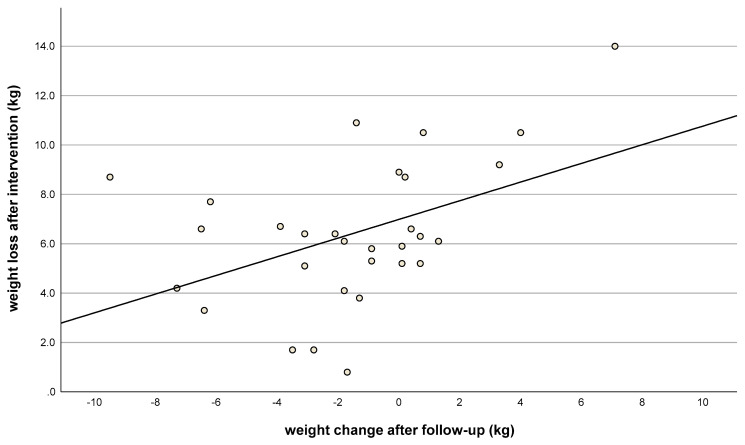
Weight loss after intervention (kg, t_1_) vs. weight change after follow-up (kg, t_2_). Correlation coefficient: r = 0.45; *n* = 30; *p* = 0.01.

In addition, greater weight loss during the intervention phase was positively correlated with greater weight loss during the follow-up ad libitum phase (r = 0.45; *p* = 0.01; Figure 3).

**Figure 4:**
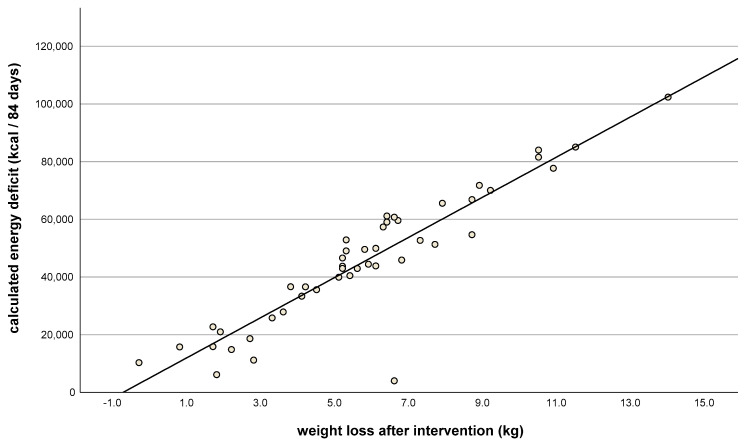
Weight loss after intervention (kg, t_1_) vs. calculated energy deficit after 84 days (kcal), as indicated by food diaries. Correlation coefficient: r = 0.9; *n* = 46; *p* < 0.001.

**Figure 5:**
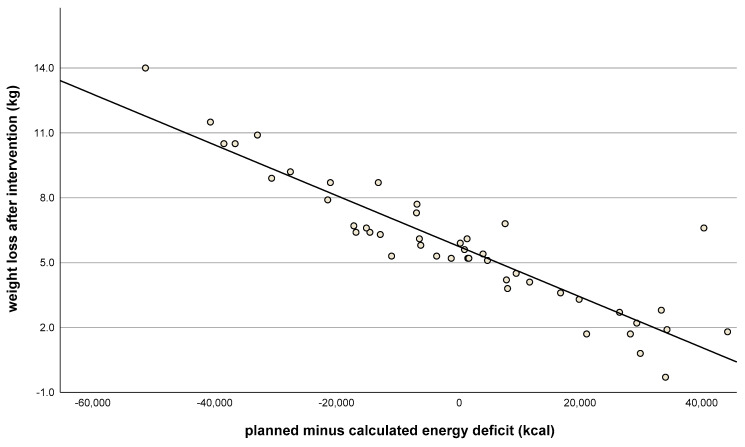
Weight loss after intervention (kg, t_1_) vs. planned minus calculated energy deficit (kcal) after 84 days (kcal), as indicated by food diaries. Correlation coefficient: r = 0.91; *n* = 46; *p* < 0.001).

The first food diary (given after the first training session) was kept and submitted by all but two of the women. The second (given after the third training session) was not submitted by four. If data from the food diaries were not available, the completed meal checklists at times corresponding to the food diaries were used as a substitute: these were available for 45 of 46 women. There were no significant differences between the real energy deficits (calculated by the food diaries) and the predicted energy deficits (calculated by the body weight planner): 6 ± 277 kcal/24 h (25 ± 1159 kJ/24 h); *p* = 0.886. Additionally, predicted individual energy deficits correlated with weight loss (r = 0.9, *p* < 0.001, Figure 4). Where there was a greater difference between the planned and real energy deficit (with the actual energy deficit lower than planned), weight loss was also lower (Figure 5). This suggests that better compliance to the energy deficit target resulted in better weight loss, closer to the desired 5–6 kg.

The participation rate in the training sessions and protocol violations (failure to adhere to the diet plan) decreased as the intervention progressed (Table 3).

The feedback of the women during face-to-face and telephone communications (Table 4) showed fewer “everything is good” statements as the intervention went on. “Fatigue” and “compliance is hard” remained at a constant level throughout. The number of “flatulence” statements was highest at the second conversation. Deviations from the food plan were clearly communicated by the women and discussed. During the follow-up women were faced with different strengths and challenges as evaluated by phone interviews (Table 4).

## 4. Discussion

The aim of this study, which was to facilitate weight loss in postmenopausal women with overweight, was fulfilled. Another positive result is the significant and lasting reduction in waist circumference. An increased waist circumference is associated with an increased risk of metabolic syndrome [31]. This was achieved via an individual approach combined with supportive group sessions. Creating individual diet plans based on each woman’s preferences and aversions, as well as the ability to change diet plans as requested during the intervention, ensured that compliance and motivation were maintained, which allowed for body weight loss. The basis for the intervention was a detailed personal diet history and monitoring of dietary patterns with a focus on different food groups. The survey of diet history was very time-consuming, but it was also very constructive and effective.

Another factor that enabled success was the close interaction between nutritionists and participants. Wishes, concerns and problems were discussed in face-to-face conversations and telephone calls, which established a relationship based on trust and mutual respect. This meant that the challenges of dieting, such as bloating, drinking shakes and maintaining motivation, were communicated and discussed honestly, and eye height protocol violations were reported, as shown by the feedback summarized in Table 4. The integration of group training sessions additionally strengthened motivation and promoted support and the exchange of experiences between peers. In their meta-analysis, Paul-Ebhohimhen et al. [32] revealed that, after 12 months, participants who attended group sessions had lost 1.4 kg more than those who attended individual counseling sessions, which highlights the importance of group training sessions. Another recent systematic review shows similar results but stresses the urgency of further studies [23].

The decreasing trend in the attendance of women at the training sessions was compensated for individual post-training meetings. Using this approach, women were empowered to change their dietary behavior and implement what they had learned in the intervention stage during the follow-up. This was shown by significant weight loss of almost 5 kg (at t_2_) on average (median 4.4 kg), suggesting that behavior change instructions given during the intervention were effective in the longer term.

Comparison of the range of weight loss, which reflects interindividual differences in response to the intervention, with previous studies remains difficult as generally only a standard deviation or confidence interval is reported in other publications. Gardner et al. [33] reported a weight change range of 40 kg (−30 kg to +10 kg) in a 12-month weight loss trial with 481 participants with overweight. In the present study, the range of weight change was relatively small at 14.3 kg (−14 kg to +0.3 kg), which illustrates the value of an individual approach in promoting weight loss in all participants.

We found an inverse association between energy intake and FFM, with rapid weight loss particularly detrimental to lean mass. Therefore, in future studies, it should be communicated to participants that losing weight too quickly could result in a high loss of FFM and muscle mass, especially in older women who have an increased risk of a decrease in FFM in contrast to an increase in absolute FM [34]. Seimon et al. [35] showed, in the 12-month TEMPO randomized trial with 85 postmenopausal women, proportional lean mass and weight loss, with an approximate 1.5-fold loss of whole-body lean mass and thigh muscle area by severe energy restriction (65–75%) compared with moderate energy restriction (minus 25–35% of total energy expenditure). In addition, a 2.5-fold greater loss of total hip bone mineral density in the severe energy restriction group illustrates another risk of fast weight loss. By contrast, a recent meta-analysis compared the effects of rapid vs. slow weight loss on body composition and REE [36]. With similar magnitudes of weight loss in both groups, FFM changes were not significantly different. However, the study indicated a greater effect on loss of FM and preservation of REE with slow weight loss compared with rapid weight loss.

In this study, an energy deficit of 750 kcal/24 kcal (circa 30%) based on total energy expenditure (TEE = REE × PAL) was targeted. This should result in an average weight loss of about 500 g per week, which was achieved by women who followed the protocol (Figure 5). We measured REE by indirect calorimetry and PAL with a 7-day activity log; the precise determination of REE and TEE is crucial to generate accurate advice on stabilizing weight. Inaccurate measurements, and therefore inaccurate energy deficit targets, can negatively influence the success of weight loss and consequently the motivation of the participants, which in turn risks the success of a weight loss intervention. To ensure energy deficit targets were met, shakes were included in the initial phase, which seemed to support weight loss and are also recommended in the German S3 guidelines [37]. The essential step is that participants learn to replace the shakes with real meals in the long term since participants reported both weight gain and increased sweet cravings after stopping shake consumption.

The success of the intervention with individual meal plans in this study is in agreement with other studies that have shown PN to be effective in promoting dietary change. In the Food4Me study [18], 1269 participants were randomized into four groups: (A) conventional dietary advice (control), (B) PN based on individual dietary intake, (C) PN based on B plus phenotypic data and (D) PN based on C plus genotypic data. Results from PN groups combined (B, C and D) showed that PN was more effective in improving dietary behaviors measured by a healthy eating index than population-based advice; however, integrating phenotypic or genotypic information did not result in further improvements. The focus of the study was not weight loss, meaning no difference in body weight was observed between groups. A meta-analysis of 13 studies also showed improved fruit and vegetable consumption in 5465 participants who received PN compared with those who received nonpersonal advice [38]. Another meta-analysis including 21 randomized controlled trials compared web-based personal interventions with nonpersonal, general information on weight loss. PN interventions were more effective, with a weighted mean difference of −1.83 kg (CIs −2.2 to −1.4 kg; *p* < 0.0001) [39]. This is why Hassapidou et al. [40] and his team incorporated the recommendation for a personalized yet flexible intervention into the European guidelines for medical nutrition therapy in adult obesity.

The delivery of information is also important in weight loss strategies: Hartmann-Boyce et al. [41] found that certain behavior change techniques promoted weight loss in adults. Strategies that were effective included those that ‘provide information about others’ approval’, ‘provide normative information about others behavior’, ‘model/demonstrate the behavior’ and ‘facilitate social comparison’. By contrast, those that ‘prompt focus on past success’ and ‘prompt self-talk’ were associated with lower effectiveness in weight loss. In accordance with this, nutritionists in the present study tried to implement effective strategies during the group sessions.

### Strengths and Limitations

The present study protocol was designed according to the recommendations of Bellg et al. [25] for enhancing treatment fidelity. The dropout rate of 15% during the intervention phase was below 20%, the latter being a value that Schulz and Grimes [42] postulate limits the validity of study results. However, during follow-up, the loss of participants was 46%. Furthermore, the number of participants in this study is limited, and there was no control group. Therefore, these results should be interpreted with caution. Further studies with a sufficient number of subjects and with a long-term follow-up period are needed. So, the results have to be verified in further randomized control studies. These results illustrate that close support is also necessary during a follow-up; a change in diet is a profound move and must be made by the person concerned. However, this is a slow process that requires a great deal of effort, and facilitation by long-term close supervision may help facilitate lasting changes. Individual approaches in combination with appreciative communication and group sessions is an involved but necessary step for this purpose. The strength of this study is that trusting relationships with the women could be established, which made care possible. This, in combination with individual meal plans, achieved excellent weight loss of 500 g per week according to guidelines [37]. This paper is a reanalysis of the main paper [24] and illustrates the positive impact of the methodological approach on weight loss in postmenopausal women. The double-blind randomized controlled trial applied an intensive assessment of diet history and individual nutritional plans, achieving valid results. Due to the a priori measurement of REE by indirect calorimetry and the assessment of PAL, a valid estimation of individual energy requirements was possible, along with an individual prescription for energy intake.

## 5. Conclusions

In conclusion, the secondary analysis of the ProSeni [24] study indicated that the described approach with a focus on nutritype combined with group sessions was an effective, though time-consuming, weight loss strategy in these postmenopausal women with overweight. The differences in diet history and resulting individual diet plans, along with the close individual and group support, were an effective way for the participants to lose weight successfully. Further studies are necessary. However, slow weight reduction and moderate energy restriction need to be explained to the participants to minimize FFM loss.

## Figures and Tables

**Table 1:** Example of daily meal schedules given after the second training session during the intervention phase.

Consecutive Meal Plan Number	Meal Overview
22	Potato salad with dill mustard sauce Pear crumble
23	Sweet potato soup served with baguette Fruit yogurt
24	Cauliflower gratin with crispy crust Fruit salad Bread with plum jam
25	Spicy mushroom rice pan Jam roll Fruit
26	Pasta cream cheese pan Marble cake
27	Macaroni in ham and leek sauce served with salad Chocolate cookie and fruit
28	Spinach and potato casserole Wild berry jelly Pretzel roll with jam
29	Potato and carrot cakes with herb curd cheese Crumble cake Fruit
30	Risotto with spinach and gorgonzola cheese Jam roll Fruit
31	Stuffed peppers with rosemary potatoes Cake

**Table 2:** Baseline characteristics and absolute changes in anthropometry, energy expenditure and blood pressure compared with preintervention (baseline; t_0_) after the weight loss intervention (t_1_) and follow-up (t_2_).

**Body Weight, kg**	**Baseline (*n* = 46)**	**Change at t_1_ (*n* = 46)**	**Change at t_2_ (*n* = 30)**
mean ± SD	83.9 ± 8.8	−5.8 ± 3.0	−1.5 ± 3.5
P ^1^		<0.001	0.024
**FFM, kg**	**Baseline (*n* = 46)**	**Change at t_1_ (*n* = 46)**	**Change at t_2_ (*n* = 30)**
mean ± SD	46.1 ± 4.8	−1.1 ± 1.2	+0.3 ± 1.5
P ^1^		<0.001	0.237
**SMM, kg**	**Baseline (*n* = 42)**	**Change at t_1_ (*n* = 42)**	**Change at t_2_ (*n* = 30)**
mean ± SD	22.4 ± 2.4	−0.8 ± 0.9	+ 0.4 ± 1.0
P ^1^		<0.001	0.063
**FM, kg**	**Baseline (*n* = 46)**	**Change at t_1_ (*n* = 46)**	**Change at t_2_ (*n* = 30)**
mean ± SD	44.9 ± 4.1	−2.7 ± 1.4	+0.7 ± 1.8
P ^1^		<0.001	0.041
**REE, kJ/24 h (kcal/24 h)**	**Baseline (*n* = 46)**	**Change at t_1_ (*n* = 46)**	**Change at t_2_ (*n* = 29)**
mean ± SD	1687 ± 181	−1096 ± 439 (−262 ± 105)	+150 ± 385 (+36 ± 92)
P ^1^		<0.001	0.042
**Waist Circumference, cm**	**Baseline (*n* = 43)**	**Change at t_1_ (*n* = 43)**	**Change at t_2_ (*n* = 27)**
mean ± SD	97.8 ± 9.4	−8.0 ± 3.2	−0.2 ± 3.4
P ^1^		<0.001	0.816
**Blood Pressure (Systole), mmHg**	**Baseline (*n* = 46)**	**Change at t_1_ (*n* = 46)**	**Change at t_2_ (*n* = 28)**
mean ± SD	137 ± 23	−12 ± 13	9 ± 11
P ^1^		<0.001	<0.001
**Blood Pressure (Diastole), mmHg**	**Baseline (*n* = 46)**	**Change at t_1_ (*n* = 46)**	**Change at t_2_ (*n* = 28)**
mean ± SD	89 ± 13	−6 ± 7	3 ± 7
P ^1^		<0.001	<0.024

^1^ Significance level for dependent t test within-group changes (t_0_–t_1_; t_1_–t_2_). FFM, Fat-free mass; SMM, skeletal muscle mass.

**Table 3:** Number of absent women and protocol violations reported at training days.

Training day	1	2	3	4
Number of absent women	1	2	7	10
Number of protocol violations	0	14	21	24

**Table 4:** Personal feedback of participants about strengths and challenges of the diet during the intervention period and follow-up phase.

First conversation		Statements							
	Strengths	*Everything is good*		*Adaptation of recipes required*			*Recipes are good*		
	*n*	5		2			2		
	Challenges	*Too much food*	*Hunger*	*Bad mood*	*Fatigue*	*Compliance*	*Boredom (with shakes)*	*Flatulence*	*Fullness*
	*n*	7	3	1	1	5	2	3	2
Second conversation		Statements							
	Strengths	*Everything is good*							
	*n*	1							
	Challenges	*Too much food*	*Hunger*			*Fatigue*	*Compliance*	*Flatulence*	
	*n*	3	3			2	2	6	
Third conversation		Statements							
	Strengths	*Everything is good*				*Belly is tighter*			
	*n*	2				1			
	Challenges	*Hunger*		*Fatigue*		*Compliance*		*Flatulence*	
	*n*	2		1		4		2	
Fourth conversation		Statements							
	Strengths	*Everything is good*		*Feels good*			*Not hungry*		
	*n*	1		1			1		
	Challenges	*Too much food*	*Hunger*	*Fatigue*		*Compliance*	*Flatulence*	*Boredom (with shakes)*	
	*n*	1	2	1		6	1	1	
	Statements during follow-up phase								
Strengths	*Would like to lose more weight*	*Trying to maintain weight loss*		*Trying to implement recommendations*		*Proud of what has been achieved*		*Doing more sports/movement*	
*n*	1	3		6		1		7	
Challenges	*Weight gain after stopping shakes*	*Increased hunger/appetite*		*Hard to maintain diet without support*		*Increased sweet cravings after stopping shakes*		*Returned to old eating behavior*	
*n*	1	2		1		1		1	

## Data Availability

Not applicable.

## Supplementary Materials

The following supporting information can be downloaded at: https://www.mdpi.com/article/10.3390/nu15143250/s1, Figure S1: Schematic representation of the study.
