# Supplementary figures and images for: Concept of an Intervention for Sustainable Weight Loss in Postmenopausal Women with Overweight—Secondary Analysis of a Randomized Dietary Intervention Study
Source: Nutrients. 2023 Jul 22;15(14):3250. doi: 10.3390/nu15143250 (PMC10383994; doi:10.3390/nu15143250)

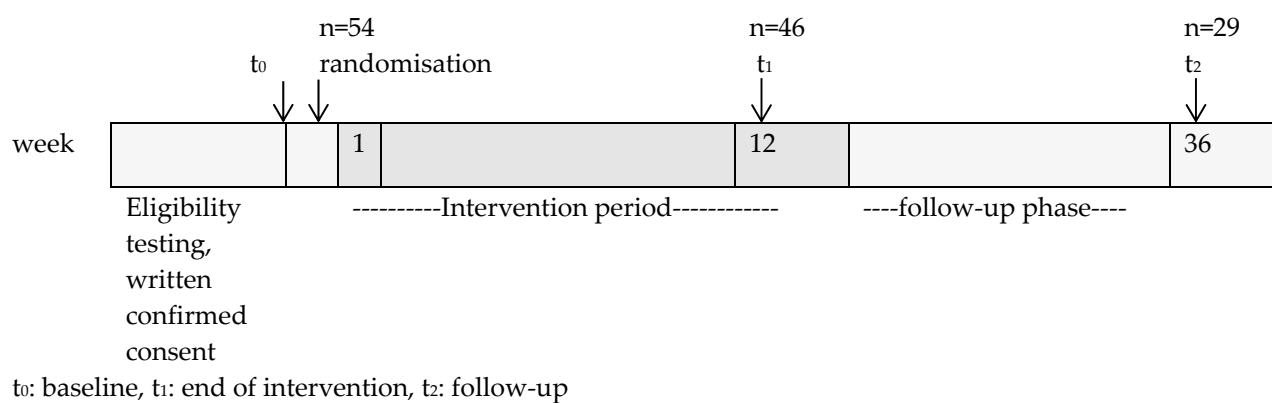

**Figure S1:** Schematic representation of the study.

Supplement: Supplementary file 1 [file nutrients-15-03250-s001.zip › nutrients-2474201-supplementary.pdf]
